# Therapeutics of Twin Pregnancies in Dairy Cattle

**DOI:** 10.3390/ani11061564

**Published:** 2021-05-27

**Authors:** Irina Garcia Ispierto

**Affiliations:** 1 Agrotecnio Center, University of Lleida, 25198 Lleida, Spain; irinag@ca.udl.cat; 2 Departmentof Animal Science, University of Lleida, 25198 Lleida, Spain

The series of eight articles (three original articles, three reviews and two com-ments) is presented by international leaders in the subject of twin pregnancies in dairy cattle. Cattle are a monotocous species, which means that under most circumstances, a successful pregnancy results in the birth of one calf. However, occasionally, the reproductive process in cattle results in the birth of twins. Twinning in dairy cows is not desirable as it can result in heavy economic losses. Based on multiple economic analyses conducted by numerous researchers, economic losses due to twinning are estimated to range between $59 to $161 per twin pregnancy [1].

Over the last 30 years, the incidence of multiple ovulations, and thus twinning, in dairy cows has increased considerably alongside milk production. Multiple ovulations are the result of the simultaneous formation of two or more codominant follicles, either from one or both ovaries. However, the final process leading to the events of ovulation can be blocked under certain circumstances, such as the development of stress. Thus, cows under stress conditions with a single preovulatory follicle at estrus may experience ovulation failure, whereas in cows with two or more co-dominant follicles, of which at least one ovulates, ovulation failure of the remaining follicles is often beyond the control of clinicians. De Rensis et al. [2] propose heat stress as a model of stress, at a time where global warming is already a serious threat to reproductive function in animals and humans. It is possible that follicular cooling before ovulation is not only susceptible to heat stress, but also to any other type of stress. Thus, the physiological pattern, or natural conditions, of the ratio between unilateral and bilateral multiple pregnancies should be close to one, as was noted during the cool period in their study. Another study found that the incidence of unilateral multiple pregnancies was higher during the warm period than the cool period, and the incidence of bilateral multiple pregnancies decreased in similar measure from the cool to the warm period [3].

For these reasons, well-managed, high-producing dairy herds need solutions to the increasing pressure to reduce the use of antibiotics and increase the lifespan of dairy cattle. The first step in the therapeutics of preventing twinning could possibly be genetic selection. McGovern et al. [4] conclude that the twinning predictions, outputted by the evaluation, effectively identify cows that are more genetically predisposed to experiencing twin pregnancies (regardless of reproduction protocol) when they enter the milking herd. Thus, including twinning in a selection index will potentially offer producers a more comprehensive tool for selecting more robust, sustainable, and profitable animals.

Taking into account the adage that “prevent is better than cure”, which is entirely appropriate for this problem, two techniques have been proposed by Garcia-Ispierto and López-Gatius [5]. The first includes transferring a single in vitro-produced embryo (IVP) to older cows, the animals with an increased risk of twin pregnancies. The second proposal includes a novel non-ultrasound guided puncture and drainage of the smaller co-dominant follicles of cows presenting more than one follicle at the time of artificial insemination (AI). To prevent ovulation failure of the non-drained follicle, HCG treatment at AI can be the best solution, even during heat stress periods [6]. Apart from eliminating twins, following GnRH treatment, five to seven days post-estrus, both procedures promote the formation of an additional corpus luteum, reducing the risk of subsequent pregnancy loss.

Whether or not this prevention is applied in farms, pregnancy diagnosis is a key point to control the likelihood of twins. The rectal palpation is able to distinguish between unilateral and bilateral twin pregnancies. Transrectal ultrasonography is the most widespread method under practical circumstances, because both unilateral and bilateral twins can be detected. Moreover, the heartbeat (and through fetal viability) and corpora lutea can be monitored. The measurement of pregnancy-specific proteins from biological fluids has been a promising tool for 20 years, but at the moment, the detection of twins is limited [7]. When twins are diagnosed on the farm during early gestation, management options might include doing nothing, terminating the pregnancy, or attempting manual embryo reduction. Induced embryo reduction is successful in multiple pregnancies in women and mares. However, in contrast to human and equine multiple pregnancies, in twin pregnant cows, vascular embryonic fusion has occurred already before the early fetal period. This important process of placental anastomosis is probably the reason for a high incidence of pregnancy loss after natural or induced reduction of twins in cattle. Methods proposed for twin reduction in cows on day 28–40 of gestation are manual crushing of the amniotic vesicle, or transvaginal ultrasound-guided suction of allanto-amniotic fluid. Before any attempt at embryo reduction, it must first be checked that both twins are alive [8]. Based on a recent economic analysis of these options, attempting manual embryo reduction decreased the economic losses of a twin pregnancy by $23 to $45 [1].

Having discussed management options after diagnosing a twin pregnancy, the best option would still be a well-designed twin pregnancy prevention program. Promising candidate strategies for this purpose include drainage without suction of co-dominant follicles at the time of insemination, the transfer of a single embryo and, of course, including twinning in a selection index.
